# Editorial for the Special Issue “Molecular Mechanisms and Innovative Therapeutic Approaches in Inflammatory Diseases, Pioneering Precision Medicine Solutions”

**DOI:** 10.3390/cimb48100999

**Published:** 2026-09-29

**Authors:** Hector A. Cabrera-Fuentes, Elisabeth Deindl, Klaus T. Preissner

**Affiliations:** 1 División de Estudios de Posgrado e Investigación, Tecnológico Nacional de México (TecNM)/Instituto Tecnológico de Tijuana, Tijuana 22414, Mexico; 2 UNAM-UABJO Research Center, Universidad Autónoma Benito Juárez de Oaxaca, Oaxaca 68120, Mexico; 3 Institute of Cardiovascular Physiology and Pathophysiology, Biomedical Center (BMC), LMU Medizin, Ludwig-Maximilians-Universität München, 82152 Planegg-Martinsried, Germany; elisabeth.deindl@med.uni-muenchen.de; 4 Institute of Surgical Research, LMU University Hospital, LMU Medizin, Ludwig-Maximilians-Universität München, 81377 Munich, Germany; 5 Department of Cardiology, Kerckhoff-Heart Research Institute, Faculty of Medicine, Justus-Liebig-University, 35392 Giessen, Germany; klaus.t.preissner@biochemie.med.uni-giessen.de

Inflammation is the common ground of a very large share of chronic human diseases, and persistent inflammatory processes substantially contribute to major causes of and the progression of morbidity and mortality worldwide [1]. Over the same period, immune-mediated inflammatory diseases affecting different organs have been increasingly understood in mechanistic terms through a limited number of signature cytokine hubs, helping explain why therapies targeting shared inflammatory pathways can be effective across distinct diseases, while individual patients may respond differently to specific cytokine-directed treatments [2]. Beneath affecting cytokines as major causal agents, mitochondrial stress and immune–metabolic reprogramming have emerged as upstream determinants of whether inflammation resolves or persists [3]. Precision medicine in inflammatory diseases therefore has three interlocking tasks: (a) to define the molecular mechanism at work in a given tissue, (b) to identify measurable markers that index the respective pathomechamism, and (c) to match such markers to respective therapies.

This Special Issue of *Current Issues in Molecular Biology*, entitled “Molecular Mechanisms and Innovative Therapeutic Approaches in Inflammatory Diseases, Pioneering Precision Medicine Solutions”, was conceived around those medicine-oriented tasks. Seventeen contributions were accepted after peer review—eleven original research articles, five reviews and one case report—with corresponding authors based in eleven countries across Asia, Europe, Africa and the Americas. The disease spectrum is wide, from the dental pulp to the hypothalamus, because the mechanisms under study are shared to a certain degree.

Four experimental studies interrogate inflammatory signalling at the cellular and tissue levels. Alrshedan et al. exposed lipoteichoic acid-stimulated human dental pulp stem cells to recombinant tumor necrosis factor superfamily member 14 (TNFSF14-LIGHT) at 25 and 50 ng/mL and found reduced tumor necrosis factor-α (TNF-α), nterleukin (IL) -6 and IL-8 together with increased IL-10 at both transcript and protein levels [4]. These findings extend the immunomodulatory repertoire of a cytokine family that is already a therapeutic target in rheumatic disease into pulpal biology [5]. Htun et al. examined caffeine, a drug given to most preterm infants for apnoea of prematurity [6], in lipopolysaccharide (LPS)-exposed THP-1 pre-monocytes, and they observed a concentration window: TNF-α transcripts fell by 71% only at 100 µM when caffeine followed LPS, whereas pre-treatment of caffeine reduced TNF-α and nuclear factor-κB (NF-κB) transcripts at all four concentrations tested, and the secreted cytokines did not change significantly at the single 2.5 h time point [7]. Inoue et al. characterized the axon-guidance cue netrin-1 and its receptors UNC5B and Neogenin-1 in a rat rotator cuff tear model (n = 10 per time point): *Ntn1*, *Unc5b* and *Neo1* transcripts rose after injury; 500 ng/mL netrin-1 transiently induced *Tnfa*, *Il6*, *Mmp1* and *Mmp3* in primary tenocytes, whereas 50 ng/mL netrin-1 enhanced early neurite extension in human induced pluripotent stem cell (iPSC)-derived sensory neurons [8], bringing to attention a molecule previously shown to retain macrophages within inflamed tissue [9]. Ammara et al. report a pilot platelet RNA-sequencing study in keratin 18–angiotensin-converting enzyme 2 (K18-ACE2) mice (ten animals in four groups) in which TNF receptor-associated factor (TRAF)-5 and TRAF6 were overexpressed after SARS-CoV-2 infection but not when infection followed chimpanzee adenovirus Oxford 1 (ChAdOx1) vaccination [10]: an observation that joins a growing literature on platelet transcriptional reprogramming in COVID-19 [11].

Two clinical studies evaluate candidate biomarkers in patients, while one case report illustrates the clinical relevance of thrombotic risk in a child with multisystem inflammatory syndrome. In 52 patients with microscopic polyangiitis, Ashida et al. measured kidney injury molecule-1 (Kim-1) in urine and in kidney biopsies and found that the two compartments index different processes: urinary Kim-1 tracked interstitial fibrosis and the Mayo Clinic Chronicity Score; tubular Kim-1 tracked crescents and the anti-neutrophil cytoplasmic antibody (ANCA) Kidney Risk Score, and neither predicted relapse, whereas C-reactive protein did [12]. That dissociation is informative in a disease in which kidney biopsy remains central to diagnosis and prognostication [13]. Matei-Man et al. followed 36 biologic-naive patients with moderate-to-severe plaque psoriasis through 12 weeks of anti-TNF, anti-IL-17 or anti-IL-23 therapy; serum TNF-α, but not IL-17A, IL-17F or IL-12/23, decreased with treatment and correlated modestly with week-12 Psoriasis Area and Severity Index (PASI) (r = 0.391), with 4.7% of variance explained by regression [14]. The small explained variance argues against TNF-α as a stand-alone monitoring marker in a disease whose treatment response is still judged by clinical scores [15]. Stoyanova et al. describe a 3-year-old boy with multisystem inflammatory syndrome in children [16], group A streptococcal co-infection and subclavian vein thrombosis on the ninth day of illness, in whom genotyping revealed Factor V Leiden, Factor V HR2 and plasminogen activator inhibitor-1 (PAI-1) 4G/5G variants [17]. Thromboembolic events are a recognized complication of this hyper-inflammatory syndrome [18], and the report argues for thrombophilia work-up when they occur in very young children.

Three articles derive candidate biomarkers from public transcriptomic data: Sun et al. combined bidirectional Mendelian randomization of 41 inflammatory cytokines with single-cell and bulk RNA sequencing of lung adenocarcinoma and nominated hepatocyte growth factor, with a genetically predicted odds ratio of 1.254 (*p* = 0.020) and a diagnostic area under the curve (AUC) of 0.872 in the primary cohort and 0.858 in an independent external cohort, together with macrophage-restricted expression [19]. Readers should note that instruments were selected at *p* < 5 × 10^−6^ and that no wet-laboratory validation was performed, two points the Strengthening the Reporting of Observational Studies in Epidemiology Using Mendelian Randomization (STROBE-MR) reporting standard asks authors to provide explicitly [20]; the inflammation-to-cancer axis under study is nonetheless mechanistically well founded [21]. Lin et al. intersected differential expression, weighted gene co-expression network analysis and least absolute shrinkage and selection operator (LASSO) regression across four Gene Expression Omnibus (GEO) datasets of Sjögren’s syndrome and periodontitis with isolated C-C motif chemokine receptor 1 (CCR1) as the single shared gene, showing AUCs of 0.74 to 0.83 across test and validation sets [22]. Cui et al. applied a comparable pipeline to inflammatory bowel disease and rheumatoid arthritis restricted to mitochondrial dysfunction-related genes, where the nominated protein disulfide isomerase family A member 4 (PDIA4) and dual specificity phosphatase 6 (DUSP6) confirmed their induction in LPS-stimulated cell lines by qRT-PCR, and reported validation AUCs of 0.664 for IBD and 0.856 for RA. In the RA validation set, DUSP6 alone reached 0.915, whereas PDIA4 amounted to 0.519 [23]. These studies are hypothesis-generating, and their value will be settled by prospective, patient-level validation of the kind formalized in the Transparent Reporting of a multivariable prediction model for Individual Prognosis Or Diagnosis + Artificial Intelligence (TRIPOD+AI) statement [24].

Two studies evaluate plant-derived interventions in dextran sulfate sodium (DSS) colitis, the most widely used chemically induced model of intestinal inflammation [25]. Zhang et al. used network pharmacology to predict complementary targets for pomegranate peel and hawthorn, quantifying synergy in LPS-stimulated RAW264.7 macrophages based on the combination index (0.69 for the extract pair and 0.245 for ellagic acid plus maslinic acid). They showed in mice that the combinations reduced serum TNF-α and IL-6, restored colonic superoxide dismutase activity, reduced p38 mitogen-activated protein kinase (p38 MAPK) and extracellular signal-regulated kinase (ERK) phosphorylation and partly normalized gut microbiota composition [26]. Choi et al. administered a chemically standardized hot-water extract of *Amomum villosum* fruit (vanillic acid 1.09 mg/g) at 100 to 500 mg/kg to mice (n = 8 per group) before and during DSS exposure and observed dose-related protection of body weight and colon length, as well as a reduction in myeloperoxidase activity, IL-6, IL-1β, TNF-α and cyclooxygenase-2 (COX-2), restored zonula occludens-1 (ZO-1) and occludin, and reduced ERK and p38 phosphorylation [27]. Both studies converge on MAPK signalling, and both leave open which constituents act in vivo, a question that recurs throughout natural-product pharmacology [28]. Mendoza-Calderón et al. address that translational gap in a semi-systematic review of thirteen plant-derived secondary metabolites in hepatocellular carcinoma, a malignancy that arises predominantly against a background of chronic inflammation [29], and provide a prioritization matrix that ranks a major component in green tea (epigallocatechingallate (EGCG)), curcumin, quercetin (an inhibitor of protein disulfide isomerase) and berberine as high-priority candidates, while stating that current evidence does not support routine clinical use [30].

Four further reviews supply mechanistic context. Gatsounia et al. compile the microRNA literature on chronic rhinosinusitis with nasal polyps, organized by molecular pathways (epithelial–mesenchymal transition, type 2 inflammation, cytokine regulation and vascular remodelling), and discuss extracellular vesicles as delivery vehicles [31]. The obstacles to RNA therapeutics they identify are those of the field at large [32]. Barone et al. review DNA methylation in Philadelphia-negative myeloproliferative neoplasms, in which global hypo-methylation is more prominent in primary myelofibrosis than in *Polycythaemia vera* or essential thrombocythaemia and in which chronic inflammation shapes the methylome [33], a perspective that complements the genetic account of these disorders [34]. Kim et al. synthesize how mitochondrial reactive oxygen species, released mitochondrial DNA acting through cyclic GMP-AMP synthase–stimulator of interferon genes (cGAS-STING) and NLR family pyrin domain containing 3 (NLRP3), and defective PTEN-induced kinase 1 (PINK1-Parkin mitophagy sustain chronic inflammation. They organize candidate interventions from mitochondria-targeted antioxidants to nicotinamide adenine dinucleotide (NAD+) precursors and mitochondrial transplantation [35]. Arias-Carrión and Ortega-Robles trace narcolepsy, modelling from canine and murine orexin-deficient lines to immune-driven and regenerative models, arguing that current models exaggerate cataplexy and under-represent narcolepsy type 2 [36]. The autoimmune hypothesis they build upon such observations rests on the detection of hypocretin-reactive T cells in patients [37], and the therapeutic horizon they describe has since been reinforced by a phase 2 trial of the orexin receptor 2 (OX2R)-selective agonist oveporexton [38].

By reading the seventeen papers and understanding their contents, several points deserve attention. The recurrent signalling nodes are the familiar ones: NF-κB, MAPK, TNF-α and IL-6 appear in nearly every experimental contribution. Such convergence favours therapeutic targeting and works against specificity, and the field needs readouts that distinguish tissue-restricted from systemic activation. Several in vitro and animal studies in this collection rely on transcript-level endpoints, limited sampling time points, single-sex cohorts, or relatively small group sizes: More complete reporting in accordance with Animal Research: Reporting of In Vivo Experiments (ARRIVE) 2.0 and pre-specified sample-size justification would strengthen the evidential weight of otherwise informative experimental studies [39], and pre-specified sample sizes would raise the evidential weight of otherwise well-designed experiments. Biomarker candidates from public datasets should be reported against the TRIPOD+AI framework [24] and confirmed in prospectively collected patient material before diagnostic language is used. Also, the discrimination measured in data that were not used for training is at the minimum, and several contributions here did meet it. Natural-product studies need chemically defined preparations, measured exposure and tests of the isolated constituents; otherwise, synergy and mechanisms remain inferred rather than explicitly demonstrated. The precision-medicine ambition of this Special Issue ultimately depends on closing the circle from marker to mechanism to therapy in the same patients; as an example, the psoriasis and vasculitis cohorts collected here show how that work begins, and how modest correlations have to be read.

None of the contributions collected here resolve inflammatory disease, and none claim to. What they offer is a set of tractable mechanisms, candidate markers with reported performance, and interventions whose limitations are, in most cases, stated by the authors themselves. We hope that the second edition of this Special Issue will continue to advance several of these lines of work toward patient-level validation. Our sincere gratitude goes to the authors for their contributions, the reviewers for their detailed and constructive evaluations, and the editorial staff of *Current Issues in Molecular Biology* for their support throughout the publication process.

## Data Availability

No new data were created or analyzed in this study. Data sharing is not applicable to this article.
